# Supplementary material for: Chromatin mapping identifies BasR, a key regulator of bacteria-triggered production of fungal secondary metabolites
Source: eLife. 2018 Oct 12;7:e40969. doi: 10.7554/eLife.40969 (PMC6234034; doi:10.7554/eLife.40969)
Supplement: Supplementary file 2. [file elife-40969-supp2.doc]

**Supplementary File 2**

List of selected genes with differentially acetylated H3K9 and different expression.

|  |  |  | **ChIP-seq** | **Microarray** |
| --- | --- | --- | --- | --- |
| **Name** | **Annotation** | **Description** | **LFC H3K9ac** | **LFC** |
| **I - Nitrogen metabolism** | |  |  |  |
| *crnA* | AN1008 | Nitrate transporter with 12 predicted *trans*-membrane domains | -1.30 | -0.01 |
| *niaD* | AN1006 | Nitrate reductase (NADPH) | -1.78 | -0.04 |
| *niiA* | AN1007 | Nitrite reductase | -1.92 | -0.14 |
| *tamA* | AN2944 | Transcriptional co-activator of the major nitrogen regulatory protein AreA | -0.80 | -0.35 |
| *gltA* | AN5134 | Glutamate synthase, NAD^+^-dependent (GOGAT) | -0.86 | -0.46 |
| *gdhA* | AN4376 | NADP-linked glutamate dehydrogenase | -1.14 | 0.06 |
| *ntpA* | AN5696 | Nitric oxide-induced nitrosothionein involved in NO detoxification | -1.16 | -0.21 |
| *meaA* | AN7463 | Major ammonium transporter | -1.22 | -0.17 |
| *ureD* | AN0232 | Nickel-binding protein involved in utilization of urea as a nitrogen source | -1.44 | -0.95 |
| *prnD* | AN1731 | Proline dehydrogenase with a predicted role in proline metabolism | -1.36 | 0.02 |
| *prnB* | AN1732 | Proline transporter | -1.39 | -0.48 |
| **II - Amino acid metabolism** | |  |  |  |
| *ugeA* | AN4727 | UDP-glucose 4-epimerase, involved in galactose metabolism | -0.82 | -0.62 |
| *qutB* | AN1137 | Quinate 5-dehydrogenase | -0.90 | 0.47 |
|  | AN9506 | Protein with predicted amino acid *trans*-membrane transporter activity | -0.98 | -0.68 |
|  | AN1923 | Putative alanine transaminase | -1.02 | 0.18 |
|  | AN5731 | Putative chorismate synthase | -1.04 | 0.03 |
|  | AN6255 | Putative cytochrome c oxidase subunit with a predicted role in energy metabolism | -1.08 | 0.35 |
|  | AN8118 | Putative cytochrome c oxidase subunit with a predicted role in energy metabolism | -1.08 | 0.33 |
|  | AN1150 | Putative transaminase with a predicted role in arginine metabolism | -0.62 | 0.10 |
|  | AN1733 | Putative delta-1-pyrroline-5-carboxylate dehydrogenase with a predicted role in glutamate and glutamine metabolism | -0.79 | -0.43 |
|  | AN2129 | Subunit 5 of the COP9 signalosome (CSN) responsible for cleaving the ubiquitin-like protein Nedd8 from cullin-RING E3 ubiquitin ligases | -0.50 | -1.16 |
|  | AN3347 | Putative amino acid transporter | -0.64 | Not detectable |
|  | AN8647 | High-affinity nitrite transporter | -0.59 | -1.96 |
|  | AN0399 | High-affinity nitrate transporter | -0.59 | 0.21 |
|  | AN0418 | Putative high-affinity urea/H^+^ symporter | -0.58 | 0.08 |
|  | AN7379 | Orthologue(s) have role in negative regulation of transcription from RNA polymerase II promoter, regulation of nitrogen utilization and nucleus localization | 0.51 | 0.33 |
| *cpcA* | AN3675 | Transcription factor of the Gcn4p c-Jun-like type | 1.02 | 1.53 |
| *jlbA* | AN1812 | bZIP transcription factor | 1.3 | 0.04 |
| **III - Calcium signalling** | |  |  |  |
|  | AN3585 | Transcript induced in response to calcium dichloride in a CrzA-dependent manner | 1.42 | 3.72 |
| *pmcB* | AN4920 | Calcium-transporting mitochondrial ATPase involved in calcium homeostasis | 1.42 | 1.66 |
|  | AN3998 | Transcript induced in response to calcium dichloride in a CrzA-dependent manner | 1.26 | 1.01 |
|  | AN3420 | Transcript induced in response to calcium dichloride in a CrzA-dependent manner | 1.05 | 1.17 |
|  | AN4418 | Transcript induced in response to calcium dichloride in a CrzA-dependent manner | 1.03 | -0.03 |
|  | AN2427 | Transcript induced in response to calcium dichloride in a CrzA-dependent manner | 1.00 | 1.07 |
|  | AN0419 | Transcript induced in response to calcium dichloride in a CrzA-dependent manner | 0.88 | -0.99 |
|  | AN3751 | Transcript induced in response to calcium dichloride in a CrzA-dependent manner | 0.86 | 3.56 |
|  | AN5372 | Transcript induced in response to calcium dichloride in a CrzA-dependent manner | 0.82 | -0.84 |
|  | AN5993 | Has domain(s) with predicted calcium binding activity | 0.76 | 8.08 |
|  | AN5341 | Orthologue(s) have calcium binding activity | 0.74 | -0.33 |
|  | AN2826 | Transcript induced in response to calcium dichloride in a CrzA-dependent manner | 0.73 | 2.12 |
|  | AN1950 | Orthologue(s) have FAD *trans*-membrane transporter activity, calcium channel activity | 0.72 | -0.26 |
|  | AN5302 | Transcript induced in response to calcium dichloride in a CrzA-dependent manner | 0.64 | 1.14 |
| *mid1* | AN8842 | Stretch-activated calcium channel | 0.63 | -0.01 |
| *pmrA* | AN7464 | Calcium-transporting ATPase with a predicted role in energy metabolism | -0.74 | -0.67 |
|  | AN8774 | Transcript induced in response to calcium dichloride in a CrzA-dependent manner | 0.55 | 1.26 |
| **IV - Development** | |  |  |  |
|  | AN4674 | Orthologue(s) have role in asexual sporulation | 1.14 | 0.28 |
| *mstC* | AN6669 | High-affinity glucose transporter active in germinating conidia | 0.92 | 1.48 |
|  | AN0928 | Orthologue(s) have role in conidiophore development | 0.90 | 0.00 |
|  | AN5619 | Orthologue(s) have metallopeptidase activity, role in ascospore development | 0.88 | 0.37 |
| *fbx15* | AN2505 | F-box protein | 0.88 | 1.25 |
|  | AN2856 | Orthologue(s) have 3'-5' exonuclease activity, role in ascospore formation, fruiting body development, pre-miRNA processing and perinuclear region of cytoplasm localization | 0.80 | 1.50 |
|  | AN3689 | Orthologue(s) have role in ascospore formation | 0.69 | 0.79 |
| *esdC* | AN9121 | Protein with a glycogen binding domain involved in sexual development | 0.68 | 0.76 |
|  | AN6898 | Orthologue(s) have role in asexual sporulation | 0.65 | -0.27 |
|  | AN1131 | Putative cytosolic Cu/Zn superoxide dismutase | 0.63 | 0.17 |
|  | AN3813 | Orthologue(s) have copper uptake *trans*-membrane transporter activity and role in aerobic respiration | 0.58 | -1.80 |
| MAT1 | AN2755 | Alpha-domain mating-type protein | 0.52 | 0.29 |
| *fcyB* | AN10767 | Purine-cytosine transporter | -0.58 | -0.57 |
| *tmpA* | AN0055 | *Trans*-membrane protein involved in regulation of conidium development | -0.58 | -3.11 |
| *fluG* | AN4819 | Cytoplasmic protein involved in regulation of conidiation and sterigmatocystin production | -0.66 | 0.43 |
| *cffA* | AN5844 | Orthologue of *Neurospora crassa* *conF*, light-induced transcript expressed during conidiation in *N. crassa* | -0.71 | 1.41 |
| *gsk3* | AN6508 | Protein kinase | -0.80 | -0.62 |
| *rasA* | AN0182 | Small monomeric GTPase of the Ras superfamily involved in regulation of development | -0.81 | -0.47 |
| **V – Secondary metabolite gene cluster** | |  |  |  |
| ***ors* gene cluster** | |  |  |  |
| *orsA* | AN7909 | Polyketide synthase | 1.57 | 6.91 |
| *orsB* | AN7911 | Putative amidohydrolase | 2.32 | 7.91 |
| *orsC* | AN7912 | Putative tyrosinase | 2.28 | 4.41 |
| *orsD* | AN7913 | Protein of unknown function | 2.64 | 4.53 |
| *orsE* | AN7914 | Putative alcohol dehydrogenase | 1.58 | 5.85 |
| ***atn* gene cluster** | |  |  |  |
| *atnK* | AN7875 | Protein of unknown function | 1.09 | 2.08 |
| *atnI* | AN7877 | RTA-like protein | 0.87 | 0.46 |
| *atnB* | AN7883 | YCII-related domain | 1.04 | 6.90 |
| *atnC* | AN11031 | Predicted transmembrane transporter | 0.69 | Not detectable |
| *atnD* | AN11028 | Predicted nucleotide binding protein with oxidoreductase activity | 0.72 | Not detectable |
| ***dba* gene cluster** | |  |  |  |
| *cipB* | AN7895 | Putative oxidoreductase | 0.89 | 3.86 |
| *dbaA* | AN7896 | Zn(II)_2_Cys_6_ transcription factor | 0.77 | 6.33 |
| *dbaB* | AN7897 | FAD-binding monooxygenase | 0.80 | 9.96 |
| **Cichorine gene cluster** | |  |  |  |
|  | AN6437 | Orthologue of *Aspergillus versicolor* Aspve1_0052718 and *Aspergillus sydowii* Aspsy1_0031878 | 1.04 | -0.39 |
|  | AN6440 | Orthologue of *Aspergillus versicolor* Aspve1_0168042, *Aspergillus sydowii* Aspsy1_0031884 | 0.76 | 3.85 |
|  | AN6441 | Protein of unknown function | 0.77 | 2.80 |
| *cicE* | AN6447 | Predicted O-methyltransferase | 0.62 | 0.18 |
| ***stc* gene cluster** | |  |  |  |
| *stcO* | AN7811 | Sterigmatocystin biosynthesis protein | 0.58 | -0.95 |
| *stcI* | AN7816 | Putative lipase/esterase | 0.59 | 0.28 |
| *stcE* | AN7821 | Norsolorinic acid reductase | 0.48 | 0.56 |
| ***mdp* gene cluster** | |  |  |  |
| *mdpD* | AN0147 | Flavin-containing monooxygenase | 0.57 | 0.19 |
| ***eas* gene cluster** | |  |  |  |
| *easC* | AN2548 | Acyltransferase | -0.58 | -3.83 |
| *easD* | AN2549 | Acyl-CoA ligase | -1.07 | -4.39 |
| **Microperfuranone gene cluster** | | | | |
|  | AN3395 | Protein of unknown function; coregulated (with *micA* and *AN3394*) | -1.18 | -1.74 |
| **VII – Transcriptional regulators** |  |  |  |  |
|  | AN0585 | Has domain(s) with predicted RNA polymerase II transcription factor activity | -0.69 | -3.12 |
| *jlbA* | AN1812 | bZIP transcription factor | 1.28 | 0.04 |
|  | AN2672 | Has domain(s) with predicted DNA binding activity | 0.81 | 0.19 |
|  | AN2839 | Has domain(s) with predicted DNA binding activity | -0.48 | 0.40 |
| *sltA* | AN2919 | C2H2 zinc-finger transcription factor | 0.81 | 0.13 |
| *tamA* | AN2944 | Transcriptional co-activator of the major nitrogen regulatory protein AreA | -0.80 | -0.35 |
|  | AN3120 | Has domain(s) with predicted DNA binding activity | 0.63 | -0.62 |
|  | AN3356 | Has domain(s) with predicted DNA binding activity | 0.87 | 1.07 |
|  | AN3433 | Has domain(s) with predicted RNA polymerase II transcription factor activity | 1.61 | 3.08 |
| *cpcA* | AN3675 | Transcription factor of the Gcn4p c-Jun-like type | 1.02 | 1.53 |
|  | AN4324 | Has domain(s) with predicted DNA binding activity | 1.41 | 0.08 |
|  | AN5052 | Has domain(s) with predicted DNA binding activity | -0.81 | 0.32 |
|  | AN6430 | Putative transcription factor with predicted role in secondary metabolite production | 1.03 | 0.83 |
| *basR* | AN7174 | Has domain(s) with predicted DNA binding activity, chromatin binding activity, similarity to yeast Bas1p | 0.64 | 5.85 |
|  | AN7765 | Has domain(s) with predicted DNA binding activity | 0.73 | 0.34 |
| *dbaA* | AN7896 | Zn(II)_2_Cys_6_ transcription factor with a role in secondary metabolite biosynthesis | 0.77 | 6.33 |
|  | AN8529 | Has domain(s) with predicted RNA polymerase II transcription factor activity | 0.49 | 0.20 |
| *zipB* | AN8772 | Putative bZIP transcription factor | 2.12 | 4.11 |
|  | AN9373 | Has domain(s) with predicted RNA polymerase II transcription factor activity | 1.22 | 0.45 |
|  | AN11165 | Has domain(s) with predicted RNA polymerase II transcription factor activity | 0.70 | 0.29 |
| *zipA* | AN11891 | Putative bZIP transcription factor | 1.04 | 0.24 |
|  | AN13001 | Has domain(s) with predicted RNA polymerase II transcription factor activity | 0.69 | Not detectable |
