# Supplementary material for: Chromatin mapping identifies BasR, a key regulator of bacteria-triggered production of fungal secondary metabolites
Source: eLife. 2018 Oct 12;7:e40969. doi: 10.7554/eLife.40969 (PMC6234034; doi:10.7554/eLife.40969)
Supplement: Supplementary file 4. [file elife-40969-supp4.doc]

**Supplementary File 5**

List of primers used in this study.

| **Name** | **Sequence (5’- 3’)** |
| --- | --- |
| **For generation of constructs for transformation of *A. nidulans*** | |
| argB2for | ATGGGAGTCAAAGTTCTGTTTGC |
| argB2rev | GGAAGCGAGAGAACATGTCAA |
| basAlbfor | GCAGATCCAATGCCAGATGC |
| basAArgBlbrev | AACAGAACTTTGACTCCCATTATGAGGAGAAGATGATTATC |
| basAArgBrbfor | GACATGTTCTCTCGCTTCCGATTACTGTGATTATTGGCAGC |
| basArbrev | AGTTAAACATCAAGGACTTGGG |
| NJ08 | TGCCAGGGATAGAAACATGT |
| NJ09 | CGCAGACCTTTCTACAGATCTGGCATATGAGGAGAAGATGATTATC |
| NJ10 | TCCGGTTTCATACACCGGGCAAAGAATCTGGACATGCGACGGAG |
| NJ11 | TCCATCTCAACTCCATCACATCACAATGACAGAACCTCGCCGG |
| NJ12 | TACCTATGTCTAGTAAAAGGAT |
| NJ41 | TTTACGGTGCACATGTTTCTATCCCTGGCACACNNNGTGTAGAAGATCTCCTACAATATTCTCAGC |
| NJ42 | CAAGAGCTATCCTTTTACTAGACATAGGTAAACTCGAGCCATCCGGAT |
| NJ102 | GCAGCTGAGAATATTGTAGGAGATCTTCTAACGCGTAGTTGCATCCATTTTCTCACTG |
| NJ103 | GATCAGGGCAAACAGAACTTTGACTCCCATGGCCTGGTGAGTTGCTTAT |
| NJ104 | AATAACTAATTGACATGTTCTCTCGCTTCCCGGCTCCATTTGTGACTGG |
| NJ105 | AACACCATATCCATCCGGATGGCTCGAGTTACGCGTTGATTGTTGTGTTGTTTTGAAGC |
| NJ106 | AACTCGAGCCATCCGGAT |
| NJ106 | TAGAAGATCTCCTACAATATTCTCAGC |
| Pabacassfor | TGCCAGATCTGTAGAAAGGTC |
| TetONfor | TCTTTGCCCGGTGTATGAAACC |
| TetONrev | TGTGATGTGATGGAGTTGAGATGG |
| TetON_pUC18tailF | cacgacgttgtaaaacgacggccagtgccatctttgcccggtgtatgaaa |
| Asyd_basRF | atggctgaacaacgtcggcg |
| Asyd_basRR | tcaatatccatacgactgcc |
| poliC_basRsidtai | agttgtccagcgccgacgttgttcagccattgtgatgtgatggagttgag |
| Ttef_sydbastail_ | ctccagagagggcagtcgtatggatattgagcggacattcgatttatgcc |
| hph_puc18tail_R | gatcctctagagtcgacctgcaggcatgcactattcctttgccctcggac |
| **qRT-PCR** | |
| Qacnfwd | CACCCTTGTTCTTGTTTTGCTC |
| Qacnrev | AAGTTCGCTTTGGCAACGC |
| qorsAfor | CTATACCACCGATAGCCAGGAC |
| qorsArev | CAGTGAGCAGGGCAAAGAAG |
| qorsDfor | GCAACGAGCCTGACATTACC |
| qorsDrev | CCGCACATCAACCATCTCTG |
| qareAfor | AAATCTAGCTCAGCGGCGAC |
| qareArev | GGGCTTTCCGCCATATCAAC |
| qniaDfor | CTGACGAAGGGGAGTGAAAG |
| qniaDrev | TCCATCCCAACGACAGTAGG |
| qprnDfor | CGCTTTTGGTCTGCGTTAC |
| qprnDrev | CCGCTCAAAAACCAGACAATC |
| qgdhAfor | TCAAGGGCATCATGGAGGAC |
| qgdhArev | CTTGGTGAAACCGGCAATG |
| qniiAfor | GCGGGAAGATGGCTGGATTTAC |
| qniiArev | CCACAGCTTCACCCTTCTTCAC |
| qtamAfor | TGATGACCAGCTCGTCAAAACC |
| qtamrev | CCGCATCGTGCATACTTTCCTC |
| qgltAfor | GCCCGTAAGAATGTCAAGACCC |
| qgltArev | GCTGAGAGCTGATGCCAGAAAG |
| qmeaAfor | TGACTACCTTGCCTGGACAC |
| qmeaArev | GCCGTTGCGATTCTTCCTTG |
| qureDfor | AGCGGGATGCAGCAAAGATG |
| qureDrev | AAGGCTCAACACTCCCAGAC |
| qprnBfor | GTCAGAGGTTGACATCTTTACG |
| qprnBrev | AAATCCACCACCAGACTCG |
| qbasRfw | GCGGGTACATGCCACAATAC |
| qbasRrev | TCTCGGGCATCATCAACTCC |
